# Salmagundi

**Published:** 1884-09

**Authors:** 


### SALMAGUNDI.

EDITED BY E. G. BETTY, D.D.S.

PROF. MAYR was present and took part in the discussions in his own peculiar manner.

DR. BARRETT exhibited some cultures of fungi made by Dr. Miller himself, which were examined with great interest.

WE don't wish to call anybody's attention to our report of the A. D. A., which appears in the next number, for—it will speak for itself.

BUGS—bugs—bugs—bugs, more bugs and toad-stools, that's what decays teeth, at least these are the common names for bacteria and fungi.

DR. ATKINSON out-Atkinsoned Atkinson, and a good many went away fully convinced that Durbin Ward is not the only man that can make a speech.

WE were very sorry that Dr. Rawls was not present to participate in the discussion of Dr. Harlan's paper on "Pyorrhœa Alveolaris," for he would have enjoyed it hugely.

WE were sorry not to see among those present the familiar countenances and genial smiles of Watt, Rehwinkle, Jennings, and "dear old Van." May his shadow never grow less nor his tailor complain of the quantity of cloth to the suit.

WE visited Dr. Atkinson at his office and saw him at work. He said that there is nothing better in the world to apply to the swollen and irritated gums, when teeth have been moved than a concentrated solution of salicylic acid in alcohol; he even furnishing patients with a portion of the solution for self-application.

WHAT was the matter with Harvard that it did not have a duly authorized representative at New York? It's possible that when the base ball season is over and the college championship settled, the powers that be may deign to think twice before it is forgotten that there *is* a dental school under the lee of the great university.

---

THE REGISTER will publish its report of the A. D. A. in the next issue, full and complete, so that its readers will get a connected idea of the proceedings in one number, as that is regarded by very many as much superior to the custom of some journals of cutting up a report and publishing a little dab every month, until, by the time the last of it is printed, readers will have forgotten what the first was.

---

THE latest improvement (?) to the remodeled Wilkerson Chair is the addition of a cushioned seat for the operator on the right side, the arm on that side being done away with to make room for the rigid frame carrying the seat. It admits of no adjustment, and being placed so far away from the head-rest is doomed to failure unless it is either put closer or provided with means by which the operator can move it to suit.

---

THE meeting of the College Faculties in New York accomplished a great deal of good work, and, judging from the harmony of desire and opinion manifested by the various gentlemen representing the colleges, we may predict that upon the foundation now laid a noble superstructure will arise that will be seen from afar off and excite the old world university "cusses" to exclaim, "What will those d——d Yankees do next?"

---

Mrs. Kingsford, an English female physician, has recently stated that M. Pasteur was not justified "in torturing thousands of animals in trying to abolish so very rare a disease as hydrophobia." Mrs. Kingsford should learn to be accurate. M. Pasteur "tortured" only forty animals. He was'nt trying to abolish hydrophobia, but to prevent its carrying off human beings. And lastly hydrophobia is not a rare disease.—[*New York Tribune.*

THE evening of the second day of the A. D. A. was devoted to a paper by Dr. J. R. Williams on "The Development of the Enamel," the main points in which were that there is no such thing as the primitive dental groove, investigators having been led into error by portions of the specimens having been lost in the preparations, and, that the enamel is developed from the outside, or in other words, the first portion formed is upon the surface of the dentine, the development progressing from that point outward until complete. His points were all handsomely illustrated by photo-micrographs projected upon a screen by the stereopticon. The whole treatment of the subject was masterly, the paper showing considerable literary ability, which, when rounded and toned by experience, will reach a high order. The evening was well spent, and in addition to new things learned, the members thoroughly enjoyed what was also in the nature of an entertainment.

---

"ONE TOUCH OF NATURE MAKES THE WHOLE WORLD KIN."—There are times in our lives when things are done that teach us there are depths in the current of human affection which are only sounded when the mute appeal of affliction opens the heart and makes us feel that its noble impulses are above price and that life with all its vicissitudes may be brightened and made precious, by the generous sympathy of our fellow-men. This was touchingly exemplified at the recent meeting at Saratoga Springs.

A bright little boy, the son of Dr. Clifton, of Waco, Texas, while playing near the entrance of Congress Park, Wednesday evening, was knocked down and run over by a heavy double team, the wheels of the carriage passing over his face and shoulder fractured the clavicle and broke the lower jaw at the symphasis. His most serious injury was the crushing of the back of his head. He was picked up and carried into the hotel, unconscious and bathed in blood. His aunt was nearly demented with grief, fearing the shock to the father who had otherwise been afflicted and whose life was wrapped up in his beautiful boy. Kind hands tenderly ministered to the needs of the injured child and gentle, subdued voices, uttered words of sympathy and hope for the future. Later in the evening when Dr. Williams' paper was concluded at the meeting, the accident was announced to the members. Vulunteers offered to act as nurses, though the Association thought it was best to procure a trained nurse, which it did at its own expense.

Before final adjournment it also instructed its treasurer to honor Dr. Clifton's draft for necessary funds. These official acts of a large body of professional men in their organized capacity, together with individual and official expressions of sympathy for the afflicted ones, is striking proof of what the human heart is capable when called upon by the urgent necessity in which calamity so often involves us when we least expect it.

'Tis acts of this nature that bring us to realize that all in this world is not lust and the greed of gain, and that there is a beauty in the manifestations of humanity that is akin to the greatness and magnanimity of the Diety.

